# Combining physical and virtual worlds for motor-cognitive training interventions: Position paper with guidelines on technology classification in movement-related research

**DOI:** 10.3389/fpsyg.2022.1009052

**Published:** 2022-12-14

**Authors:** Luka Šlosar, Claudia Voelcker-Rehage, Armin H. Paravlić, Ensar Abazovic, Eling D. de Bruin, Uros Marusic

**Affiliations:** ^1^Science and Research Centre Koper, Institute for Kinesiology Research, Koper, Slovenia; ^2^Department of Health Sciences, Alma Mater Europaea – ECM, Maribor, Slovenia; ^3^Neuromotor Behavior and Exercise, Institute of Sport and Exercise Sciences, University of Münster, Münster, Germany; ^4^Faculty of Sport, University of Ljubljana, Ljubljana, Slovenia; ^5^Faculty of Sports Studies, Masaryk University, Brno, Czechia; ^6^Faculty of Sport and Physical Education, University of Sarajevo, Sarajevo, Bosnia and Herzegovina; ^7^Department of Neurobiology, Care Sciences and Society, Karolinska Institute, Stockholm, Sweden; ^8^Department of Health, OST – Eastern Swiss University of Applied Sciences, St. Gallen, Switzerland; ^9^Department of Health Sciences and Technology, Institute of Human Movement Sciences and Sport, ETH Zurich, Zurich, Switzerland

**Keywords:** extended reality, virtual reality, augmented reality, mixed reality, exergaming, taxonomy, classification

## Abstract

Efficient movements require intact motor and cognitive function. There is a growing literature on motor-cognitive interventions to improve the overall quality of life of healthy or diseased older people. For such interventions, novel technological advances are crucial not only in terms of motivation but also to improve the user experience in a multi-stimuli world, usually offered as a mixture of real and virtual environments. This article provides a classification system for movement-related research dealing with motor-cognitive interventions performed in different extents of a virtual environment. The classification is divided into three categories: (a) type of digital device with the associated degree of immersiveness provided; (b) presence or absence of a human-computer interaction; and (c) activity engagement during training, defined by activity >1.5 Metabolic Equivalent of task. Since virtual reality (VR) often categorizes different technologies under the same term, we propose a taxonomy of digital devices ranging from computer monitors and projectors to head-mounted VR technology. All immersive technologies that have developed rapidly in recent years are grouped under the umbrella term Extended Reality (XR). These include augmented reality (AR), mixed reality (MR), and VR, as well as all technologies that have yet to be developed. This technology has potential not only for gaming and entertainment, but also for research, motor-cognitive training programs, rehabilitation, telemedicine, etc. This position paper provides definitions, recommendations, and guidelines for future movement-related interventions based on digital devices, human-computer interactions, and physical engagement to use terms more consistently and contribute to a clearer understanding of their implications.

## Introduction

With rapid technological development, the technologies used by the gaming sphere (Koivisto and Hamari, 2019) have become more affordable, easier to use and also more commonly used for research purposes. They not only allow the design of virtual spaces and stimuli but also a cost-efficient and almost precise tracking of body parts (full-body kinematics). This development is of particular interest for movement-related interventions in exercise and health settings (Skjæret et al., 2016) as it offers the opportunity for enhancing physical strength and overall engagement in physical activity (Liberatore and Wagner, 2021).

Due to the rapid development and widespread use of technologies, differences in their definition and naming have emerged, making it difficult to compare different applications or assess their impact through systematic reviews and meta-analyzes. For example, the term virtual reality (VR) has become a general term for a variety of forms of pure motor, pure cognitive, or motor-cognitive exercises that include computerized animations ranging from the projection of two-dimensional (2D) virtual realities on computer and TV screens and (curved) projections to head-mounted displays (HMDs) offering a realistic three-dimensional (3D) viewing experience (Roettl and Terlutter, 2018). Furthermore, the user and technical system interaction is diverse, ranging from computer mouse manipulation (i.e., 2D user interface) to a more natural interaction with cameras, depth sensors and haptic or tactile feedback. The diverse digital-based technologies used have a strong influence on the user’s experience and underlying psychophysiological mechanisms, modulated by multiple aspects such as first or third person feedback, the type and range of movements possible to control the action. The degree of VR immersion and embodiment (i.e., self-representation in VR) therefore have a strong influence on the positive (e.g., motor and/or cognitive rehabilitation) and negative (cybersickness) outcomes of the intervention.

The discrepancy in naming technologies has led us to write this position paper with the aim to (1) promote discussion and provide optimal taxonomy with recommendations on the emerging topic of technology integration in exercise interventions, and (2) to point to the original research needed to further develop this field (Europe, 2002; De Boeck et al., 2014; McCaskey et al., 2018). We describe and categorize some of the technologies used and dimensions (motor/cognitive) involved, with a special focus on movement-related devices that target both physical and cognitive functioning.

We must note that there have been several other attempts providing similar yet boundary definitions often referring to the same content but lumping different terms together (Mann et al., 2018; Speicher et al., 2019; Skarbez et al., 2021). We herewith update these attempts and go beyond earlier approaches as none of them incorporated human-computer interaction (HCI) and physical activity levels to provide a comprehensive definition of terms.

To guide the discussion on the emerging topic of technology integration into exercise interventions and offer recommendations for necessary original research, we first propose a taxonomy and corresponding definitions for future movement-related interventions based on the digital device used, user-related experience, and physical engagement level to use terms more consistently. We herewith aim to facilitate reading literature, searching, and effect summarizing (e.g., in systematic reviews and meta-analyzes). In the second step, we discuss and provide some recent advances in the gaming industry that have evolved from computer games to exergaming and, hence, to various motor-cognitive activities performed using extended reality (XR) as potential tools for research, motor-cognitive training programs, rehabilitation, telemedicine, and more. By providing a graphical scheme with the generic term XR and its subcategories, we hope to further stimulate the discussion and its use in the scientific literature.

## Definitions

**Figure 1:**
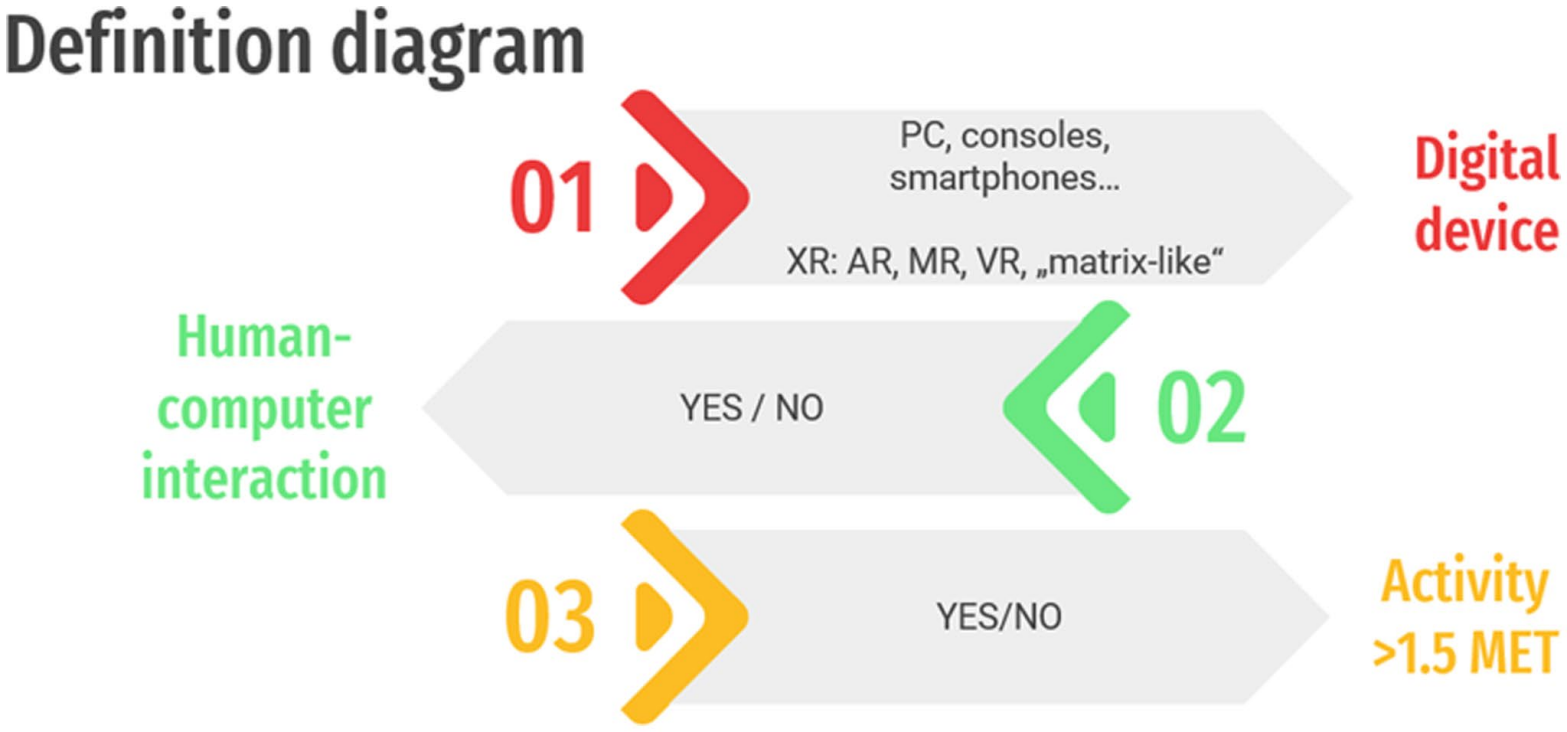
Definition diagram of technology used in movement-related research. XR, Extended Reality; AR, Augmented Reality with semi- and fully-immersive options; MR, Mixed Reality, VR, Virtual Reality; “Matrix-like VR” refers to a fully immersive technology that will be developed in the future and, as illustrated in Skarbez et al. (2021), involves a discontinuity between external virtual environments and the right-end anchor, “Matrix-like” VR.

First, we propose categories and definitions of technologies and devices used in movement-related research. As it is shown in the scheme on technology used in movement-related research (Figure 1), categorization can be performed based on (01) the digital device used and consequently the immersiveness/virtuality levels, (02) the presence, or absence of a HCI, as well as (03) the level of physical engagement.

### 1st category: Digital device

In the literature, VR is a term most often used to describe different types of intervention that use different digital devices, but hardly anyone knows what they exactly mean. A viewpoint and taxonomy close to ours has been presented in a recent paper by Skarbez et al. (2021). This paper builds on Milgram and Kishino’s reality-virtuality continuum concept (Milgram and Kishino, 1994), where we have a purely real environment on one side and a purely virtual environment on the other. This continuum concept was based on the technology available at that time, especially virtual displays, including PCs, consoles, and tablets. A recently proposed revised version of the reality-virtuality continuum considers how the interoceptive and exteroceptive senses are stimulated by technology and the continuum of the model was extended from the “external” (real) and virtual environment to “matrix-like virtual environment” (i.e., a fully immersive technology able to stimulate both the interoceptive and exteroceptive senses). State-of-the-art digital devices do not support complete control over the interoceptive senses, making the “matrix-like virtual environment” a purely theoretical concept. Conversely, our model focuses on the existing systems, classified upon the technology used to incorporate the digital content within the user’s physical reality which determines the level of immersiveness. The following paragraphs, therefore, provide an overview of the technology used with PC, consoles, tablets, and other smart technologies as well as XR technologies, including AR, MR, and VR.

#### PC and consoles with displays and controlling gadgets

The first category used to describe the classification is the presence of a digital device manipulated by the user. Early examples of interventions used a PC (Coleman and Harry, 2004), which were later to some extent transferred to various consoles (Šlosar et al., 2021), tablets (Rand et al., 2015) and other smart technologies (Gagliardi et al., 2018). Initial gaming and indeed early research by use of this technology were conducted in a seated position, with a computer monitor or projector displaying a 2D or 3D view in front of the user. The degree of immersiveness was rather low as peripheral vision or field of view was allowed outside the projected task (i.e., outside the display area).

With the introduction of motion-sensitive input devices such as Wii and Kinect, aspects of HCI interaction evolved, ranging from computer/virtual games with static sitting postures to exergames (a combination of physical activity and game mechanics supported by digital technologies) with TV, computer or (huge/curved) projector screens with cameras creating avatars that move in synchrony with the user, and therefore increase engagement with the game and/or intended task. Recently, the visual context was cleverly projected onto the treadmill surface (van Ooijen et al., 2016) aiming to elicit task-specific gait adjustments. A treadmill can be further integrated in a motion frame with a up to 240° cylindrical projection screen (the GRAIL system – Gait Real-time Analysis Interactive Lab). Despite the technical innovation, both interventions are categorized as “PC category” due to their lower degree of immersiveness – the virtual environment does not fully (360°) surround the user.

Head-mounted immersive technologies are the latest display devices that have been developed. In the following, we defined all (currently available) immersive technologies that have been developed remarkably fast in recent years and are grouped under the umbrella term XR. The idea of XR is to either combine virtual and real environments or to create a fully immersive user experience generated by computers and wearable technologies. Nowadays, XR is an emerging term for immersive technologies that are already in use, such as augmented reality (AR), mixed reality (MR) and virtual reality (VR) and others that are still in development.

#### Augmented realities

The term AR as we know and use it today stands for applications/devices that overlay computer-generated content (e.g., graphics, audio, location data) onto the real world that can superficially interact with the environment in real-time. The AR user can interact and manipulate the virtual world by adding or removing aspects of the real world. However, the virtual content is dictated by the software platform which cannot significantly change its structure when the user performs body movements. For example, in a Moving through Glass AR application (Tunur et al., 2020), users are allowed to evoke virtual trainers that, however, cannot recognize the user’s body movements and respond accordingly. ARcore and ARkit exemplify Google and Apple’s respective platforms for creating AR experiences (Concha et al., 2021), which enable more complex AR software solutions in conjunction with devices such as smartphones, tablets, smart glasses, and headsets. Unlike other XR technologies, AR can be experienced in a fully- or semi-immersive viewing environment. Since the degree of immersiveness has a major impact on the user’s sensorimotor experience (Perez-Marcos, 2018), the present classification model distinguishes between the semi-immersive (ARs) and fully immersive (ARf) AR environments. In the former, virtual enhancements (e.g., auditory, visual, or other sensory input) are integrated with real-world objects perceived in 2D. For a fully immersive AR experience AR glasses or HMDs are needed to create a stereoscopic 3D effect.

#### Mixed realities

The ideology behind MR is to use an overlay of synthetic content that is anchored to and interacts with objects in the real world and real-time. MR is often understood as AR with additional graphical interfaces, but the two terms should be distinguished due to the complex technical requirements in software development as well as the user experience. In AR, virtual content usually appears as visual information that is superimposed into the user’s field of view and acts in a user-independent manner (Sauer et al., 2017). According to the development advances of MR, virtual objects are not only integrated into the physical world, but the user can interact with the virtual content to change the nature of the objects in the physical world. This object manipulation can takes place in a hybrid environment of real and virtual worlds, which enables a new experience through gaze, gesture, and speech recognition technology. Examples of MR include products such as Bridge (Occipital) and HoloLens (Microsoft), which allow users to map any space and place computer-generated objects within it (Kress, 2020).

#### Virtual realities

In contrast to the aforementioned, VR technology allows users to be fully immersed in a purely synthetic (computer-generated) digital environment, that completely replaces the physical world. Current applications of VR offer a variety of HMDs, e.g., Oculus (Facebook), the HTC-Vive series, and Valve Index (Steam), providing a 360-degree view of an artificial world that tricks the brain into thinking these are fully immersive experiences. Users can interact with the virtual world *via* remote controllers, haptic feedback devices, and their own body gestures captured by motion-tracking technologies. The user’s visual and haptic perceptions are fully perceived and transferred to the virtual environment. It is important to note that these technologies (e.g., Oculus) can also be used for MR experiences where the virtual environment is superimposed on the real environment, which is possible through the frontal camera. Such an approach can be found in a recently developed immersive VR experience called Cave Automatic Virtual Environment (CAVE), a room-sized immersive 3D visualization system developed to overcome the problems of HMDs, such as limited mobility, especially for movement-related research applications (Gromer et al., 2018). According to many authors (Mütterlein and Hess, 2017; Mazikowski, 2018), CAVE systems offer the most immersive installations of VR. Further 3D-visualization systems that allows real-time moving (walking) through a 360° immersive 3D visualization has been developed (CAREN System - Computer Assisted Rehabilitation Environment). Therefore, a 3-axis dual-belt treadmill or a motion platform with six degrees of freedom is integrated in a motion frame system with an up to 360° cylindrical projection screen and surrounded sound. It allows to program challenging environments that mimic real-life scenarios using advanced virtual and augmented reality.

Following this digital device classification might help to prevent misclassification of interventions. For example, studies that use motion-sensing devices to transmit and display body gestures to a screen in real-time would not be classified as VR. Choi and Lee’s (2019) kayak paddling, for example, cannot be interpreted as a VR intervention because the visual context was projected onto a screen and thus did not fully surround the user. Similarly, Thielbar et al. (2020) and Kim et al. (2019) use the terms VR therapy and VR training to describe the intervention in which users control an avatar *via* the Kinect (Microsoft) and Wii (Nintendo) systems. Although a virtual environment is certainly present in all these studies, the user interaction and visual experience differ from the VR description of the interventions presented above. Since the users were not completely (360°) surrounded by the virtual environment, nor were they able to overlay real objects with the computer-generated ones, as is the case in AR and MR, these interventions would be classified as PC according to our categorization. To facilitate the distinction between the presented digital devices, in Table 1 we provide a short description with the main characteristics of each.

**Table 1:** A short distinction between the terms used to classify digital devices.

PC	VR	AR	MR
A purely synthetic virtual environment projected on a screen, TV, or any other device (e.g., treadmill) that does not completely (360°) surround the user.	Immerses users in a fully digital artificial environment where they are no longer able to see the real world. Perfect in cases where you want to train or view a totally different world.	Overlays virtual content on a real-world environment, either perceived in 2D or 3D. Very useful when you still want to be able to see the real world.	Overlays virtual content on a real world environment, but the virtual content also interacts with objects in the real world. AR can be seen as a subcategory of Mixed Reality.

### 2nd category: Human-computer interaction

The second classification can be made through the presence or absence of HCI. Generally, HCI is an interdisciplinary field of study that examines the interface between a digital device and the user. Human capabilities, goal-directed experiences, graphical interfaces, and context are the main HCI components (Hochheiser and Valdez, 2020). Although each of these components requires specific mention, further discussion of each component is beyond the scope of this paper, which focuses solely on the context related to communication between the user and the device. To simplify the classification system, interaction is confirmed (yes/no) when the digital device recognizes human body gestures and movements and provides immediate and interactive feedback (Yang et al., 2009). The distinguishing element of immediate and interactive feedback comprises most digital-based interventions (DBI), whether they are classified as PC or XR. However, the way body gestures and movements are captured varies depending on the digital device used. Interaction can occur with a fixed joystick (Oh et al., 2019), handheld controllers with gyroscopes and accelerometers, e.g., Wii Remote and PlayStation Move (Williamson et al., 2013) that allow free movement within defined boundaries, and motion capture technologies with depth sensors (e.g., Microsoft Kinect, GRAIL, CAREN) capable of tracking human skeleton data, posing, and inserting the user into different dimensions within the VR environment (Regazzoni et al., 2014). It is important to point out that the use of HMDs does not in itself imply interaction. Although the sense of presence when viewing a video from the first-person perspective is strong, the intervention does not lead to interaction in the absence of virtual embodiment, i.e., self-representation in VR (Perez-Marcos, 2018) and motion-induced feedback mechanisms.

The potential of sensors capable of assessing psychophysiological responses to tailor the user experience and feedback has recently been used in the rehabilitation of people with disabilities (Al-Qaysi et al., 2021) and monitors physiological responses of the user, e.g., heart rate variability (Rodriguez et al., 2021), heart rate, skin temperature, emotional arousal (Šlosar et al., 2021). DBI represents a safe and controllable application field to test the benefits of physiological HCI. Since this work focuses on the presence or absence of a HCI, further discussion of this topic is beyond the scope of this paper.

### 3rd category: Physical activity

Beside the digital device and the presence of HCI, interventions can be further classified by the amount of physical activity involved. This can either be described/differentiated by the body parts involved, the intensity levels, or by the types of exercises performed during the intervention. Especially in the field of mobility rehabilitation, certain therapies using digital devices are performed in a sitting position or on ergometers (cycle, rowing treadmill). For example, a patient’s energy output while performing tasks in a pure sitting position is lower than when performing the same task on a cycle ergometer or treadmill. Although, strongly advisable, specific intensity level measurements are often not provided by the researchers. Therewith, the physical activity classification will be simplified according to whether the task is performed dynamically or in a sitting position. Interventions, therefore, will be recognized as dynamic when the energy expenditure exceeds that derived from several common sedentary behaviors (Oh and Yang, 2010), set at 1.5 Metabolic Equivalent (MET) of task (Mansoubi et al., 2015).

Although not included in our classification system, we are aware that, in addition to physical exertion, the type of motor activity associated with cognitive stimulation must also be considered and further assessed using a multi-dimensional analysis (Torre and Temprado, 2022) to assess the efficacy of DBI compared with more conventional type training interventions. As this position paper does not specialise in different interactive constructs, we believe that further investigations of the mechanisms underlying DBI would benefit from bringing together the aforementioned criteria.

### Category: Exergames

A particular type of dynamic intervention is exergames. The development of exergames came to mass media attention in the late 1990s with the release of Konami’s Dance Dance Revolution (Tan et al., 2002) and Nintendo’s Wii in the early 2000s (Graves et al., 2007). Since then, these video games have been widely used not only for recreation, but adjusted for potential rehabilitation (Garcia-Agundez et al., 2019; García-Bravo et al., 2021) and therapeutic use (Fang et al., 2019; Pacheco et al., 2020). The widespread interest for custom-designed exergames in rehabilitation and therapeutic practices (Vallejo et al., 2020) within the rapidly evolving game interfaces development, created perplexity in defining the right terminology. The American College of Sports Medicine [ACSM] (2013) defines exergaming as: “technology-driven physical activities, such as video game play, that requires participants to be physically active or exercise to play the game. These games require the user to apply full body motion to participate in virtual sports, in group fitness exercise or other interactive physical activities.” Although the ACSM classification is widely used in the literature, the authors’ interpretation often remains vague. For this reason, we will extract the most important aspects of this definition (digital device used, immersiveness and physical engagement) and expand on them.

## Recommendations and categorization

**Figure 2:**
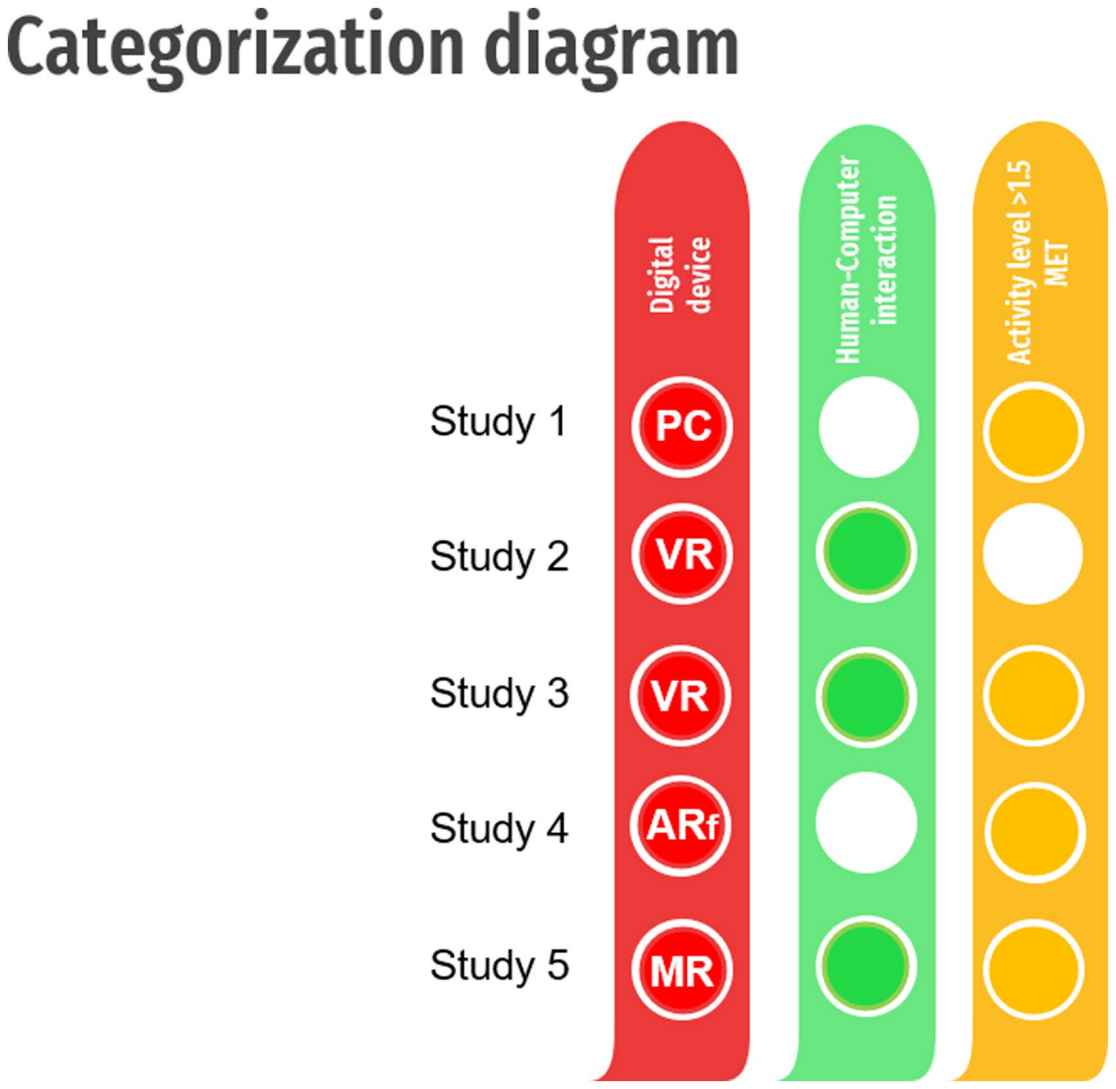
Categorization diagram of technology used in movement-related research. PC – personal computers and consoles with displays and controlling gadgets; XR, Extended Reality; AR, Augmented Reality with semi – (ARs) and fully-immersive (ARf) options; MR, Mixed Reality, VR, Virtual Reality; the white circles indicate no HCI or physical activity level below 1.5 MET.

To make a clear distinction between the effects, the present paper introduces a classification system (Figure 2) based on the following three key points:

- 1st Red column – Digital device
  According to the definitions provided in the 1st Category section, the Digital device is categorized as PC, VR, AR semi-immersive, AR fully immersive, or MR.
- 2nd Green column – Human-computer interaction
  Following the present paper classification model, the interaction is confirmed or rejected, based on the given description in the 2nd Category section.
- 3rd Yellow column – Physical activity
  The intervention is classified as dynamic when the energy expenditure exceeds 1.5 MET. Whenever the intensity level is not provided, the classification is simplified in terms of whether the intervention comprised a sitting workstation, required a standing/stepping position, or no physical activity is required.
- + Category – Exergame
  Whenever a digital device is defined, an interaction confirmed, and the energy expenditure set as higher than 1.5 MET, the intervention can be further recognized as an exergame.

An example of such classification is provided by Figure 2. Additionally, a sample model for meta-analysis and systematic reviews is provided in Supplementary material.

*Study 1* (Cho and Lee, 2013)

- *Red column.* Patient walking on a treadmill, with a TV screen positioned in front: classified as PC.
- *Green column.* No interaction confirmed (according to the following description: “The treadmill speed and the VRRW scene movement were not synchronized”)
- *Yellow column.* Physical activity higher than 1.5 MET (according to the following description: “All subjects walked on a motorized treadmill”)

*Study 2* (Song and Lee, 2021)

- *Red column.* Tasks to improve upper limb function using HMD: classified as VR.
- *Green column.* An interaction confirmed (according to the following description: “VR content consisted of rehabilitation tasks to improve upper limb function”)
- *Yellow column.* Physical activity lower than 1.5 MET (according to the following description: “All contents were configured to be performed while sitting”)

*Study 3* (Erhardsson et al., 2020)

- *Red column.* Playing VR commercial games using HMD: classified as VR.
- *Green column.* An interaction confirmed (according to the following description: “The gameplay allow individuals to play through attachment of the hand controller”)
- *Yellow column.* Physical activity higher than 1.5 MET (according to the following description: “Beat Saber (a rhythm-based game) was overwhelmingly the most common game to see play”
  + The intervention is additionally classified as an exergame.

*Study 4* (Tunur et al., 2020)

- *Red column.* AR-based dance application called “Moving through Dance” (MTG) designed for Google Glass: classified as ARf.
- *Green column.* No interaction confirmed (the glasses can be activated by voice, but the evoked virtual images do not interact with the user)
- *Yellow column.* Physical activity higher than 1.5 MET (according to the following description: “The MTG modules used in this study were designed to promote adherence to dance-centered physical activity in individuals with PD”)

*Study 5* (Glueck and Han, 2020)

- *Red column.* MR action game training with the Microsoft HoloLens platform: classified as MR.
- *Green column.* An interaction confirmed (according to the following description: “the player must avoid fire (by ducking and dodging) and “blast” opponents by directing their gaze and using a finger tap response to faire a blaster”)
- *Yellow column.* Physical activity higher than 1.5 MET (according to the same description as in the Green column)
  + The intervention is additionally classified as an exergame.

## Conclusion

Future technology will certainly bring advances and optimization of the user experience to XR and beyond. The concepts presented in this paper are relevant to the gaming experience, but also open up perspectives for research opportunities and applications in sports, rehabilitation, (tele)medicine, military training, and many other fields. In attempting to create an umbrella term for a better classification of studies involving PC, AR, VR, and MR technologies, our intention was not to criticize previous reports but, on the contrary, to create an easily understandable taxonomy dealing with different technologies that have been developed recently and will continue to expand. In the last decade, there have been several studies proposing their own XR taxonomies (Normand et al., 2012; Mann et al., 2018; Speiginer and Maclntyre, 2018; Speicher et al., 2019) and researchers who reinterpreted Milgram and Kishino’s reality-virtuality continuum (Skarbez et al., 2021). While these studies contribute to certifying boundaries between the real and virtual environments with possible hybrid conjunctions, none of them incorporates HCI and levels of physical activity to provide a comprehensive definition of technologies. We also provide a user-friendly categorization diagram and clarify the necessary characteristics for exergame interventions. We hope to stimulate further discussion and better definitions, as well as the use of the XR taxonomy currently used in scientific publications, especially in the fields of aging, sports, rehabilitation, (tele)medicine, and related fields.

As mentioned in Section 2.3, this work does not cover specific interactive constructs related to the HCI and physical activity dimensions but focuses on providing a general taxonomy upon which various frameworks can be built upon to provide clearer insight into the underlying DBI mechanisms. In comparison to previous research on functional aspects (of the motor and cognitive components) of interventions (Temprado, 2021; Torre and Temprado, 2022), we propose a taxonomy built on different extents of a virtual environment that modulates user immersiveness and virtual embodiment. Both in turn relate to the HCI and physical activity dimensions of our taxonomy, which need to be further conceptualized within the proposed classification system to improve user experience and DBI efficiency.

Future growth of the gaming market is expected along with further development, optimization and minimization of the technologies and sensors used (Parekh et al., 2020). An increase in scientific publications using this technology is also expected. Our unified classification system, therefore, provides the opportunity to more optimally classify movement-related research that uses a digital device to transport purpose developed exercise content. In this way, the implications arising from research using such technology will be better understood, more easily quantified, and translated into everyday practice.

## Author contributions

LŠ and UM contributed to the writing of the first draft of the manuscript. All authors contributed to subsequent revisions and approved the final manuscript.

## Funding

This study was supported by the European Union’s Horizon 2020 research and innovation program under grant agreement no. 952401 (TwinBrain – TWINning the BRAIN with machine learning for neuro-muscular efficiency). The authors also acknowledge financial support from the Slovenian Research Agency (research core funding no. P5-0381) and by the Deutsche Forschungsgemeinschaft (DFG, German Research Foundation) – Project-ID 416228727 – SFB 1410.

## Conflict of interest

The authors declare that the research was conducted in the absence of any commercial or financial relationships that could be construed as a potential conflict of interest.

## Publisher’s note

All claims expressed in this article are solely those of the authors and do not necessarily represent those of their affiliated organizations, or those of the publisher, the editors and the reviewers. Any product that may be evaluated in this article, or claim that may be made by its manufacturer, is not guaranteed or endorsed by the publisher.
